# Chick Hairy1 protein interacts with Sap18, a component of the Sin3/HDAC transcriptional repressor complex

**DOI:** 10.1186/1471-213X-7-83

**Published:** 2007-07-10

**Authors:** Caroline J Sheeba, Isabel Palmeirim, Raquel P Andrade

**Affiliations:** 1 Life and Health Sciences Research Institute (ICVS), School of Health Sciences, University of Minho, 4710-057 Braga, Portugal

## Abstract

**Background:**

The vertebrate adult axial skeleton, trunk and limb skeletal muscles and dermis of the back all arise from early embryonic structures called somites. Somites are symmetrically positioned flanking the embryo axial structures (neural tube and notochord) and are periodically formed in a anterior-posterior direction from the presomitic mesoderm. The time required to form a somite pair is constant and species-specific. This extraordinary periodicity is proposed to depend on an underlying somitogenesis molecular clock, firstly evidenced by the cyclic expression of the chick *hairy1 *gene in the unsegmented presomitic mesoderm with a 90 min periodicity, corresponding to the time required to form a somite pair in the chick embryo. The number of *hairy1 *oscillations at any given moment is proposed to provide the cell with both temporal and positional information along the embryo's anterior-posterior axis. Nevertheless, how this is accomplished and what biological processes are involved is still unknown. Aiming at understanding the molecular events triggered by the somitogenesis clock Hairy1 protein, we have employed the yeast two-hybrid system to identify Hairy1 interaction partners.

**Results:**

Sap18, an adaptor molecule of the Sin3/HDAC transcriptional repressor complex, was found to interact with the C-terminal portion of the Hairy1 protein in a yeast two-hybrid assay and the Hairy1/Sap18 interaction was independently confirmed by co-immunoprecipitation experiments. We have characterized the expression patterns of both *sap18 *and *sin3a *genes during chick embryo development, using *in situ *hybridization experiments. We found that both *sap18 *and s*in3a *expression patterns co-localize *in vivo *with *hairy1 *expression domains in chick rostral presomitic mesoderm and caudal region of somites.

**Conclusion:**

Hairy1 belongs to the hairy-enhancer-of-split family of transcriptional repressor proteins. Our results indicate that during chick somitogenesis Hairy1 may mediate gene transcriptional repression by recruiting the Sin3/HDAC complex, through a direct interaction with the Sap18 adaptor molecule. Moreover, since *sap18 *and *sin3a *are not expressed in the PSM territory where *hairy1 *presents cyclic expression, our study strongly points to different roles for Hairy1 throughout the PSM and in the prospective somite and caudal region of already formed somites.

## Background

The adult vertebrate body is composed of various segmented structures, such as the vertebrae, ribs, intervertebral disks, peripheral nerves and skeletal muscles. One of the earliest manifestations of segmental patterning during vertebrate embryogenesis is the generation of transient, serially repeated structures known as somites. Somites are epithelial blocks of paraxial mesoderm cells that are laid symmetrically on either side of the neural tube from the rostral extremity of the presomitic mesoderm (PSM) in an anterior-posterior (AP) manner. Ongoing gastrulation at the posterior part of the PSM ensures embryo growth as new somite pairs arise from the anterior PSM. These structures are periodically formed and the time required to form a somite pair, as well as the total number of somite pairs generated, is extraordinarily constant and species-specific. In the chick embryo a new pair of somites is formed every 90 min, to a total of 52 somite pairs [1,2].

In 1997, Palmeirim and collaborators disclosed a molecular clock underlying vertebrate embryo somitogenesis when analysing the expression pattern of the *hairy1 *gene in chick embryos [3]. This gene is cyclically expressed in PSM cells every 90 min, which corresponds to the time required for a new somite pair to be generated [3]. The number of *hairy1 *oscillations at any given moment is proposed to provide cells with temporal and positional information along both anterior-posterior and medial-lateral PSM axes [4]. Further work performed in our lab showed that a molecular clock is also operating during limb bud development [5], suggesting that the segmentation clock is not an exclusive property of PSM tissue, but may be a more general way to count time during vertebrate development, providing positional information to different types of cells.

Besides participating in the somitogenesis clock in PSM cells, *hairy1 *is continuously expressed in the caudal region of formed somites. Newly formed somites present two distinct compartments, which are the basis for the resegmentation process that later leads to vertebrae formation [6]. Somite AP polarity is generated in the anterior PSM by Mesp2 and Delta/Notch signalling [7] and the maintenance of rostral and caudal identities in newly formed somites is tightly dependent on *tbx18 *and *uncx4.1 *gene expression, respectively [8-10]. The regionalized, persistent *hairy1 *expression in the posterior somite region suggests that Hairy1 could participate in somite polarity establishment and/or maintenance, although this has not yet been addressed experimentally.

Hairy1 belongs to the hairy-enhancer-of-split (Hairy E(Spl)/HES) family of transcriptional repressor proteins [11]. All HES members share conserved functional motifs: the basic domain (b) required for DNA-binding, a helix-loop-helix dimerization domain (HLH), an orange domain (OR) which confers specificity among family members and the C-terminal WRPW tetrapeptide required for co-factor binding [12,13]. The evidence to date suggests that HES proteins can be involved in an array of repression mechanisms *via *the recruitment of different protein partners, forming complexes with different specificities [14]. HES-mediated transcriptional repression has been shown to be an important feature of many embryonic developmental processes [15-17]. Data obtained from mouse and zebrafish embryos have shown that Hairy-related proteins act as transcriptional repressors in somitogenesis: *hes1 *participates in auto-regulatory negative feedback loops [18], probably mediated by direct protein interaction with the Notch signalling component RBP-Jκ[19]; *hes7 *represses transcription from N box- and E box-containing promoters [20]; *hes7 *represses its own transcription and also *Lfng *expression [21,22]; Her13.2 interacts with Her1 and represses both *her1 *and *her7 *[23].

Although the expression of chick *hairy1 *and its homologues has been demonstrated during somitogenesis in many animal models, it is not clear what the outputs of *hairy1 *expression are, both in the molecular clock and during somite formation and differentiation. Chick Hairy1 is only known to form homo-dimers and hetero-dimers with two other HES family members, Hey 1 and Hey2, through their bHLH-OR protein domains [24]. Furthermore, the mechanisms of Hairy1-mediated transcriptional repression have not yet been addressed in the chick embryo. Aiming at understanding the molecular events triggered by chick Hairy1, we have employed the yeast two-hybrid system to identify Hairy1 protein interaction partners. The yeast two-hybrid system [25] is a well established technique used to study protein-protein interactions, which are critical in virtually all cellular processes. Sap18 (Sin3-associated polypeptide, 18 kDa), a component of the Sin3/histone deacetylase (HDAC) transcriptional repressor complex [26], was found to interact with the C-terminal portion of the Hairy1 protein. Hairy1/Sap18 interaction was independently confirmed by *in vitro *co-immunoprecipitation assays. Whole mount *in situ *hybridization experiments showed that *sap18*, *sin3a *and *hairy1 *mRNA co-localize in the rostral-most part of the chick embryo PSM and in the caudal compartment of already formed somites. Our results indicate that in these tissues chick Hairy1 may mediate gene transcriptional repression by recruiting the Sin3/HDAC complex through a direct interaction with the Sap18 adaptor molecule. Moreover, since *sap18 *and *sin3a *are not expressed in the PSM territory where *hairy1 *presents cyclic expression, our study strongly points to different roles for Hairy1 throughout the PSM and in the prospective somite and caudal region of already formed somites.

## Results

### Interaction of Hairy1 with Sap18 revealed by yeast two-hybrid analysis

**Figure 1:**
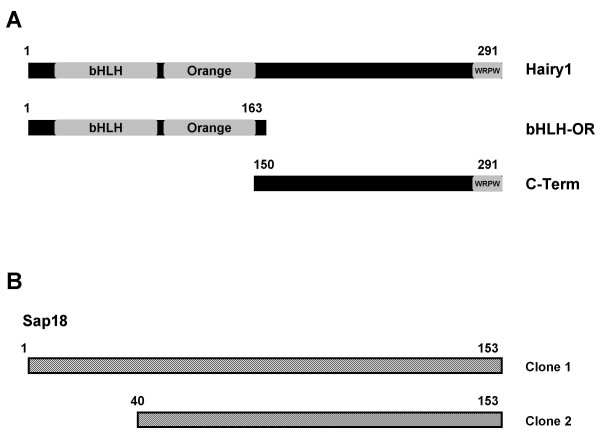
**Schematic representation of Hairy1 protein domains assayed for interaction and Sap18 clones obtained from the yeast two-hybrid screen**. (A) Hairy1 protein domains used as *bait *in the yeast two-hybrid assays; (B) Yeast two-hybrid positive interaction clones encoding for Sap18. Numbers correspond to amino acids in (A) and (B).

The yeast two-hybrid system was used to screen proteins that associate with the C-terminal portion of the chick Hairy1 protein (Fig. 1A; C-Term, amino acids 150–291). mRNA was extracted from stage HH20 – HH22 chick embryos and a cDNA library was constructed in the pGADT7 plasmid. The C-Term fragment of the Hairy1 protein was cloned in the pGBKT7 vector and the resulting *bait *construct was used to screen the constructed chick embryo cDNA library. Two different plasmids were isolated from positive-interaction yeast colonies which coded for the entire chick Sap18 protein (Fig. 1B; clone 1) or a truncated form, lacking the first 39 amino acids (Fig. 1B; clone 2).

**Figure 2:**
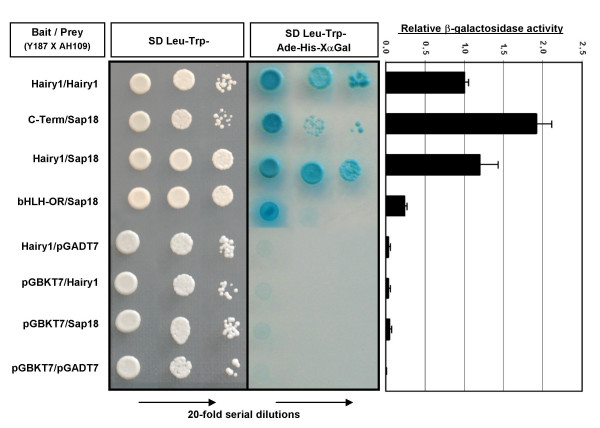
**Hairy1 and Sap18 protein interaction evaluated by the yeast two-hybrid assay**. *Baits *were expressed as Gal4 DNA-BD fusion proteins in the pGBKT7 plasmid and transformed into the Y187 yeast strain; *preys *were expressed as Gal4 AD fusion proteins in pGADT7 vector and transformed into the AH109 strain. *Bait*- and *prey*-transformed strains were mated and the resulting diploids were cultured in SD-Leu/-Trp medium, selecting for the presence of both plasmids and also in SD-Leu/-Trp/-His/-Ade/X-α-gal medium, were only the diploids presenting protein interactions are capable of growth. 20-fold serial dilutions were performed prior to colony plating to ensure growth on the selective medium is dependent solely on *HIS3*, *ADE2 *and *MEL1 *reporter gene expression. β-galactosidase activity was assayed for each strain and is presented relative to that obtained for the Hairy1/Hairy1 interaction. In all assays, Hairy1/Hairy1 protein interaction was used as a positive control and the empty vectors were employed in the negative controls. (DNA-BD: DNA binding domain; AD: activation domain).

Hairy1 protein interaction with Sap18 was confirmed by performing additional yeast two-hybrid experiments using the whole Hairy1 protein, or its N-terminus (Fig.1A; bHLH-OR, amino acids 1–163) as *bait*. The documented interaction between Hairy1 proteins in a homodimer [24] was used as a positive control (Fig. 2). *Bait *and *prey *constructs were transformed into the opposite mating-type yeast strains Y187 and AH109, respectively, and the transformed strains were mated and spread on appropriate selective media. Positive interactions were observed for Hairy1/Hairy1, C-Term/Sap18 and Hairy1/Sap18, while no interaction was detected between bHLH-OR/Sap18 or in the negative control reactions, as expected (Fig. 2).

The relative strength of Hairy1/Sap18 protein interaction was determined by scoring for reporter β-galactosidase activity (Fig. 2), using the Hairy1/Hairy1 protein interaction as a reference [24]. We found that Hairy1 has a similar affinity for Sap18 as it has for Hairy1 itself, and that the association between Hairy1 C-terminal portion and Sap18 is almost twice as strong as that for the whole protein (Fig.2). This is most probably due to the absence of the DNA-binding bHLH domain, leading to a relief of reporter gene repression. Altogether, our yeast two-hybrid results indicate that Hairy1 associates with Sap18 and that Hairy1's C-terminal domain mediates this interaction.

### Co-immunoprecipitation of Hairy1 with Sap18

**Figure 3:**
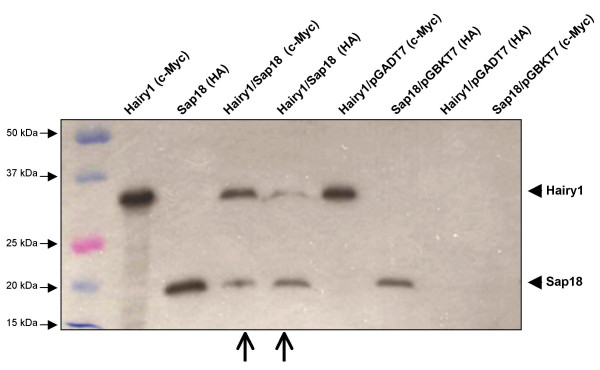
***In vitro *co-immunoprecipitation of Hairy1 and Sap18**. The proteins tested are indicated on top of each lane, as well as the antibody used for immunoprecipitation in each assay (in brackets). Proteins produced form the pGADT7 and pGBKT7 empty plasmids were used in negative controls. Open arrows indicate the gel lanes where both Hairy1 and Sap18 proteins are observed, which is indicative of a protein interaction among them.

To independently validate the interaction between chick Hairy1 and Sap18 proteins, *in vitro *co-immunoprecipitation assays were performed. Chick *hairy1 *and *sap18 *genes cloned in pGBKT7 and pGADT7 vectors, respectively, were used to generate ^35^S-labeled c-Myc-Hairy1 and HA-Sap18 through *in vitro *transcription/translation reactions. Upon incubation of both proteins, Hairy1 immunoprecipitated with Sap18 when either c-Myc or HA antibodies were employed (Fig. 3). This was not the case when *in vitro *transcription/translation reaction products of the empty vectors were used. This experiment confirms the Hairy1/Sap18 protein association identified in the yeast two-hybrid assay.

### Characterization of chick *sap18 *mRNA expression pattern

Having established that chick proteins Hairy1 and Sap18 can interact, we set out to determine if this protein interaction could be of biological relevance in our system, namely, during the process of somitogenesis. If that were so, *sap18 *gene expression should co-localize with *hairy1 *mRNA distribution in the chick embryo.

**Figure 4:**
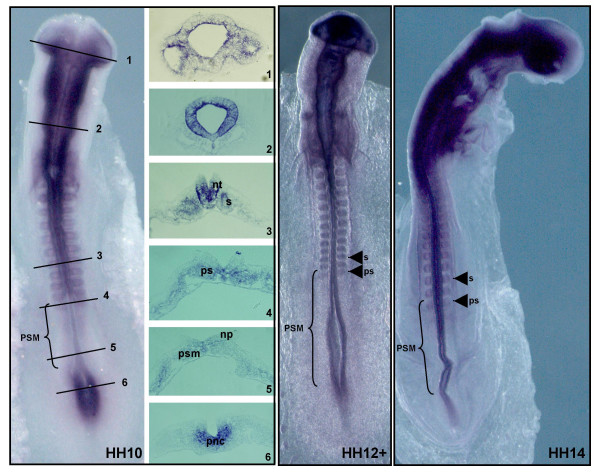
***sap18 *mRNA expression pattern in chick embryos**. Chick embryos staged HH10, HH12+ and HH14 were processed for *in situ *hybridization using a probe for chick *sap18*. Transverse sections at the indicated AP levels on stage HH10 embryo are shown. At early stages, *sap18 *is expressed at the level of the primitive streak in prospective neural cells (section 6, pnc), is absent from the neural plate (np) facing the PSM (section 5, psm) and is upregulated throughout all the neural tube positioned rostrally to the PSM territory (sections 1–3, nt). In older embryos, *sap18 *is expressed along the entire neural tube AP axis (stages HH12+ and HH14). In all embryos, *sap18 *expression is absent from most of the PSM tissue (brackets) being upregulated only in the rostral part of the PSM, the region that will give rise to the prospective somites (whole embryos and section 4, ps) and in already formed somites (whole embryos and section 3, s).

A *sap18 *antisense mRNA probe was generated and whole-mount *in situ *hybridization experiments were performed with the purpose of characterizing *sap18 *expression pattern in the chick embryo. At early stages, *sap18 *is expressed at the level of the primitive streak in prospective neural cells (Fig. 4, section 6), is absent from the neural tube facing the PSM (Fig. 4, section 5) and is upregulated throughout all the neural tube positioned rostrally to the PSM territory (Fig.4, sections 1–3). In older embryos, *sap18 *is expressed along the entire neural tube AP axis (Fig.4, HH12+ and HH14). In all embryos, *sap18 *expression is absent from most of the PSM tissue, being upregulated only in the rostral part of the PSM, the region that will give rise to the prospective somites, and in already formed somites. Chick *hairy1 *is also present in the prospective somites and in the caudal region of formed somites [3], indicating that the Hairy1/Sap18 protein interaction might play a functional role in these tissues.

### Co-localization of *hairy1, sap18 *and *sin3a *mRNA expression patterns

Sap18 has been shown to interact directly with Sin3a and act as an adaptor molecule bridging the Sin3/HDAC complex to sequence-specific DNA-binding proteins [26]. Since Sap18 associates with Hairy1, it could be recruiting the Sin3a complex for Hairy1-mediated transcriptional repression during chick embryo development. For this to be true, both *sap18 *and *sin3a *mRNAs should co-localize with each other and with *hairy1 *gene expression domains.

**Figure 5:**
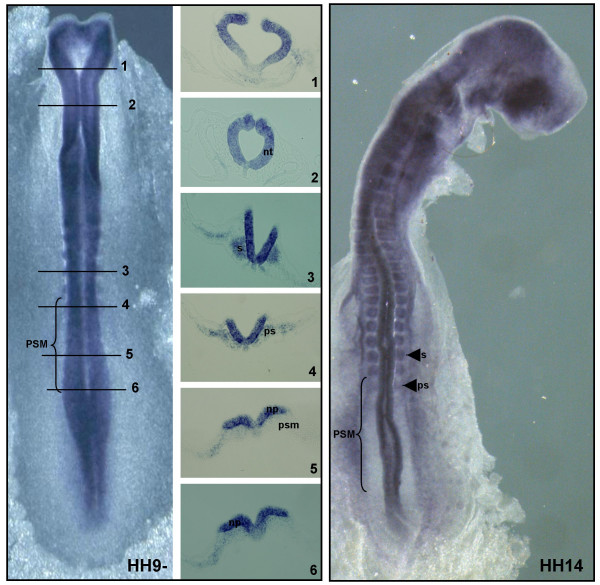
***sin3a *mRNA expression pattern in chick embryos**. Chick embryos staged HH9- and HH14 were processed for *in situ *hybridization using a probe for chick *sin3a*. Transverse sections at the indicated AP levels on stage HH9- embryo are shown. In all embryos, *sin3a *is expressed in the neural plate (np) and along the entire neural tube AP axis (nt), is absent from the PSM (section 5, psm; brackets in whole embryos) and is upregulated in the rostral part of the PSM, the region that will give rise to the prospective somites (whole embryos and section 4, ps) and in already formed somites (whole embryos and section 3, s).

**Figure 6:**
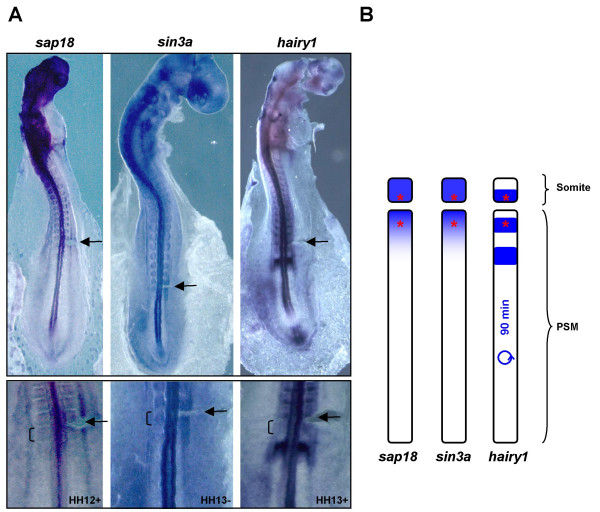
**Co-localization of *hairy1*, *sap18*and *sin3a *mRNA expression in the prospective somitic territory and somites**. (A) Whole mount *in situ *hybridization with the probe indicated on top was performed in chick embryos in somitogenesis developmental stages (upper panel). The lower panel is an amplified view of the rostral PSM and somites. Prior to *in situ *hybridization processing, a slit was made on the right side of the embryo (arrow), in the boundary between the last formed somite and the rostral PSM, evidencing the prospective somitic territory (bracket). (B) Schematic representation of the genes' expression domains, indicating (with an asterisk) the prospective somite and caudal region of already formed somites, where *hairy1*, *sap18 *and *sin3a *mRNA expression co-localize. Cyclic expression of *hairy1 *with a 90 minute periodicity is indicated.

A *sin3a *antisense mRNA probe was generated and whole-mount *in situ *hybridization experiments revealed that many *sin3a *expression domains (Fig. 5) coincide with those of *sap18 *in the chick embryo, namely in the prospective somite region and in somites (Fig. 6). This is in accordance to their reported protein interaction and biological role in transcriptional regulation. Moreover, both *sap18 *and *sin3a *expression patterns co-localize with *hairy1 *mRNA in the caudal region of somites and in the rostral part of the PSM (Fig. 6). These results suggest that in these tissues Hairy1/Sap18 interaction could be mediating the recruitment of the Sin3/HDAC repressor complex to Hairy1-target gene promoters.

Although *hairy1 *expression can be observed throughout the whole chick PSM, both *sap18 *and *sin3a *mRNAs are absent, except in the rostral-most region. This implies that Hairy1 is not capable of exerting gene transcriptional repression through Sap18-mediated Sin3/HDAC complex recruitment in the PSM regions where it is cyclically expressed.

## Discussion

Members of the hairy-enhancer-of-split (Hairy E(Spl)/HES) family of proteins are reported to be transcriptional repressors. HES proteins can form homo- or hetero-dimers and repress gene expression either through direct binding to *cis *regulatory elements in the promoter or by sequestering, and thereby inactivating, transcriptional activators of the target gene. When bound to promoter DNA, HES proteins can recruit transcriptional regulatory protein complexes through their C-terminal domain and therefore exert repressor activity (reviewed by [13]).

**Figure 7:**
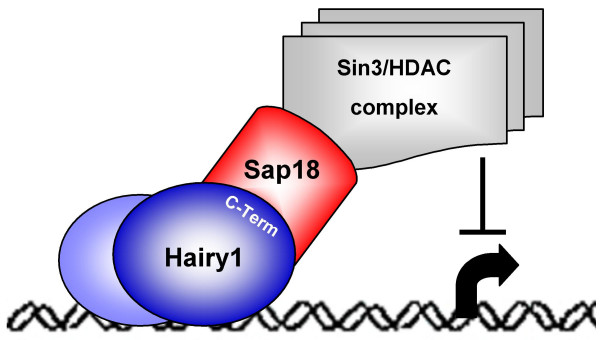
**Proposed model for Hairy1-mediated transcriptional repression in the prospective somite and caudal region of somites**. In the prospective somite and caudal region of already formed somites, where *sap18*, *sin3a *and *hairy1 *expression co-localize, a homo- or hetero-dimer containing Hairy1, bound to specific promoter regulatory sequences, may interact with Sap18 through the Hairy1 C-terminal domain and recruit the Sin3/HDAC complex to the transcriptional site, thus repressing target gene expression.

This study reports a protein interaction screen performed with the C-terminal portion of the chick hairy-enhancer-of-split protein Hairy1, using yeast two-hybrid technology. We have established that Sap18, the 18-kDa Sin3-associated protein, is a Hairy1-interacting protein, using both yeast two-hybrid experiments and *in vitro *co-immunoprecipitation assays. Chick embryo *sap18 *and *sin3a *expression patterns were characterized for the first time and were found to co-localize with each other and with *hairy1 *mRNA in the prospective somitic territory and in the caudal region of already formed somites. Our results provide novel insights into Hairy1-mediated transcriptional repression during chick embryo development, suggesting that Hairy1 may repress target gene transcription through a direct interaction with Sap18 protein, recruiting the Sin3/HDAC transcriptional repressor protein complex (Fig. 7).

The Sin3/HDAC complex is highly conserved from yeast to human and contains HDAC activity, causing transcriptional repression mainly by deacetylating histones (reviewed by [27]). Sap18 is an integral member of the Sin3/HDAC transcriptional repressor complex and it functions as an adaptor polypeptide, tethering the protein complex to promoters by interacting with sequence-specific DNA binding proteins, since members of the Sin3/HDAC complex lack intrinsic DNA binding activity [26,28]. To date, the mechanisms of Hairy1-mediated transcriptional repression have not been addressed in the chick embryo. Our work suggests that upon binding to specific DNA promoter elements, chick Hairy1 is capable of interacting with Sap18, which in turn recruits the Sin3/HDAC repressor complex, repressing Hairy1-target gene transcription (Fig. 7). In fact, the recruitment of the Sin3/HDAC complex through interaction with Sap18 has already been shown for the *Drosophila *GAGA factor [29] and Bicoid genes [30], as well as for mouse suppressor of fused protein, mSu(fu) [28] and DNA polymerase, DPE2 [31].

The gene encoding Hairy1 presents cyclic expression in the chick embryo PSM, while in somites *hairy1 *expression ceases to cycle and is persistent in the posterior somitic region [3]. In their recent review on the HES gene family, Kageyama and collaborators [13] point out that low levels of *Hes1 *down-regulate cell cycle inhibitors, promoting cellular proliferation. On the other hand, continuous and high levels of *Hes1 *expression inhibit the cell cycle. This highlights another, as yet, unaddressed issue in somitogenesis: Is Hairy1 involved in the same biological functions in the PSM, where it is cyclically expressed in undifferentiated mesenchymal cells, and in the caudal somitic region, where its expression is persistent?

Our study strongly points to different roles for Hairy1 in the PSM cyclic expression domain and in the rostral-most PSM and caudal region of somites. The expression of both *sap18 *and *sin3a *genes is absent from PSM cells and is up-regulated only in prospective and already-formed somites. This clearly indicates that Hairy1 is only capable of interacting with Sap18, recruiting the Sin3/HDAC complex for transcriptional repression in these tissues and not along the entire PSM, suggesting that this mechanism is not involved in the somitogenesis clock machinery. Instead, we can hypothesize that Hairy1 transcriptional repression through Sap18-mediated Sin3/HDAC complex recruitment may be required for somite formation and/or polarization.

## Conclusion

We have identified an interaction between the C-terminal domain of the chick protein Hairy1 and the Sin3/HDAC repressor complex adaptor molecule, Sap18. The expression patterns of chick *sap18 *and *sin3a *genes were characterized for the first time and were shown to co-localize with *hairy1 *mRNA in the rostral PSM and caudal region of formed somites. Our results indicate that Hairy1 may be mediating transcriptional repression in these tissues by interacting with Sap18 and recruiting the Sin3/HDAC transcriptional repressor complex to the promoters of its target genes (Fig. 7). Moreover, since *sap18 *and *sin3a *are not expressed in the PSM territory where *hairy1 *presents cyclic expression, our study strongly points to different roles for Hairy1 throughout the PSM and in the prospective somite and caudal region of already formed somites.

## Methods

### Eggs and embryos

Fertilised chick (*Gallus gallus*) eggs obtained from commercial sources were incubated at 37.2°C in a 49% humidified atmosphere and staged according to the Hamburger and Hamilton (HH) classification [32].

### Yeast two-hybrid assays

Chick embryo cDNA library construction and yeast two-hybrid screening were performed using the Matchmaker Library Construction & Screening Kit (Clontech, USA). A cDNA library in the pGADT7-Rec (*prey*) vector was constructed using RNA extracted from the forelimbs of chick embryos in stages HH 20 – HH22. PCR-generated *hairy1 *fragments were cloned in the pGBKT7 plasmid for preparation of the *bait *vectors. The primers used were: Hairy1 – forward: 5'ATGCCCGCCGACACGGGCATG3', reverse: 5'CTACCACGGCCGCCAGACG3'; C-Term – forward: 5'CAGATCGTGGCCATGAACTACCTGC3', reverse: 5'CTACCACGGCCGCCAGACG3'; bHLH-OR – forward: 5'ATGCCCGCCGACACGGGCATG3', reverse: 5'GCCAGCAGGAGGGGGTGGCAG3'. Plasmid construction was confirmed upon sequencing.

The haploid yeast strain Y187 was transformed with the *bait *vectors and tested for toxicity. The haploid AH109 strain was transformed with the generated pGADT7-chick embryo cDNA library. Positive and negative protein interaction controls supplied with the kit were performed with the expected outcomes. Yeast screening for potential interacting proteins with the C-terminal domain of Hairy1 protein was performed by mating Y187 transformed with pGBKT7+C-Term and AH109 transformed with pGADT7-cDNA library. Diploids were plated on SD-Leu/-Trp/-His medium and growing colonies, indicative of a potentially positive interaction between the *bait *(C-Term) and the *prey *(library protein), were re-streaked several times on SD-Leu/-Trp/-His/-Ade plates. Finally, the positive clones were replica-plated on maximally selective SD-Leu/-Trp/-His/-Ade/X-α-gal medium to ensure that colonies contained the correct phenotype. Colony PCR using primers flanking the multiple cloning site of the pGADT7 plasmid was performed on the colonies that successfully passed the reporter tests. Clones presenting an insert over 300 bp were harvested for plasmid isolation and sequencing.

To confirm Hairy1 interaction with Sap18, yeast Y187 was transformed with *bait *vectors while AH109 was transformed with *prey *plasmids. After mating, the diploid strains were cultured in SD-Leu/-Trp medium and in SD-Leu/-Trp/-His/-Ade/X-α-gal medium. Diploids were suspended in sterile water and three 20-fold serial dilutions were performed, after which a drop of each suspension was placed on each medium. This procedure ensures that the growth observed is not due to feeding on underlying cells, but can clearly be attributed to reporter gene expression. Hairy1/Hairy1 protein interaction was used as a positive control and the empty vectors were employed in the negative controls.

### Quantification of β-galactosidase activity

Quantification of reporter β-galactosidase activity was performed using the Yeast β-Galactosidase Assay Kit (Pierce, USA). Four co-transformed colonies of each combination were grown in liquid SD medium lacking leucine and tryptophan, assayed in triplicate and the mean value was calculated. Hairy1/Hairy1 protein interaction was used as a positive control and the empty vectors were employed in the negative controls. Mean activity values were normalized to those obtained for the Hairy1/Hairy1 protein interaction.

### *In vitro *co-immunoprecipitation assays

Chick *hairy1 *and *sap18 *genes cloned in pGBKT7 and pGADT7 vectors, respectively, were used to generate ^35^S-labeled Hairy1 fused to the c-Myc epitope and Sap18 fused to the HA epitope using the TnT Quick Coupled Transcription/Translation System (Promega, USA). *In vitro *co-immunoprecipitation assays were performed using the Matchmaker Co-IP Kit (Clontech, USA) following the manufacturers instructions. Samples were run on 12 % SDS-PAGE gel and the gel was dried at 80°C under constant vacuum and exposed to an X-ray film for autoradiography. Empty vectors were employed in the negative controls.

### *In situ *hybridisation probes

*In situ *hybridisation probes for *sap18 *and *sin3a *were generated. Reverse transcription and polymerase chain reactions (RT-PCR) were used to isolate chick *sap18 *(Genbank: NM_204312) and portions of *sin3a *(Genbank: XM_413695) using the sense oligos 5'-CCGGAATTCATGGCGGTGGAGTCGCG-3', 5'-TGAAGACTCCACTTTTGTGAGC-3', and antisense oligos 5'-CCGCTCGAGTCAGTATGGCCTCATGCGGC-3', 5'-TGTACCAGTTGCTGTTCACG-3', respectively. The DNA fragments generated were cloned into the pCR^®^II-TOPO^® ^vector (Invitrogen, USA) and plasmid DNA was isolated. The constructs were confirmed upon sequencing. Digoxigenin-labelled RNA probes were obtained from linearised plasmids, according to standard procedures.*hairy1 *probe generation was performed as previously described [3].

### Embryo whole mount *in situ *hybridisation and cryosectioning

Embryos were fixed overnight at 4°C in 4% formaldehyde with 2 mM ethylene glycol-bis (β-amino-ethyl ether) tetraacetic acid (EGTA) in PBS, rinsed in PBT (PBS, 0,1% Tween 20), dehydrated in methanol and stored at -20°C. Whole mount *in situ *hybridisation was performed according to the previously described procedure [33].

Some *in situ *hybridized embryos were processed for cryosectioning as previously described [34]. 12 μm cryostat (Leica CM1900) sections were collected on Superfrost Plus Slides (Menzel-Glasse). Slides were mounted in Aquatex (Merck, Germany) and photographed.

### Imaging

Embryos processed for *in situ *hybridization were photographed in PBT/0.1% azide, using a Leica DFC320 digital camera coupled to a Leica MZFLIII stereomicroscope equipped with Leica IM50 image manager (Leica Microsystems, Germany). Cryosectioned embryo sections were photographed using an Olympus DP70 camera coupled to an Olympus BX61 microscope.

## Authors' contributions

CJS prepared the *sin3a *probe plasmid, synthesized the probes, staged and collected the chick embryos, performed *in situ *hybridizations, performed the yeast two-hybrid confirmations, β-galactosidase and co-immunoprecipitation experiments and assisted in manuscript writing; IP contributed to experimental design and interpretation of results, was involved in the conception and coordination of this project and assisted in manuscript preparation; RPA prepared all yeast two-hybrid plasmids plus the *sap18 *probe vector, constructed the chick embryo cDNA library in pGADT7, performed the yeast-two hybrid screen, contributed to experimental design and interpretation of results, was involved in the conception and coordination of this project and wrote the manuscript. All authors approved the final manuscript.
